# Effects of Hydroxyurea on Skeletal Muscle Energetics and Function in a Mildly Anemic Mouse Model

**DOI:** 10.3389/fphys.2022.915640

**Published:** 2022-06-15

**Authors:** Constance P. Michel, Laurent A. Messonnier, Benoit Giannesini, Benjamin Chatel, Christophe Vilmen, Yann Le Fur, David Bendahan

**Affiliations:** ^1^ CRMBM, CNRS, Aix Marseille University, Marseille, France; ^2^ Laboratoire Interuniversitaire de Biologie de la Motricité, Université Savoie Mont Blanc, Chambéry, France

**Keywords:** hydroxyurea, anemia, skeletal muscle function, skeletal muscle energetics, magnetic resonance imaging, magnetic resonance spectroscopy

## Abstract

Hydroxyurea (HU) is a ribonucleotide reductase inhibitor most commonly used as a therapeutic agent in sickle cell disease (SCD) with the aim of reducing the risk of vaso-occlusion and improving oxygen transport to tissues. Previous studies suggest that HU may be even beneficial in mild anemia. However, the corresponding effects on skeletal muscle energetics and function have never been reported in such a mild anemia model. Seventeen mildly anemic HbAA Townes mice were subjected to a standardized rest-stimulation (transcutaneous stimulation)-protocol while muscle energetics using ^31^Phosphorus magnetic resonance spectroscopy and muscle force production were assessed and recorded. Eight mice were supplemented with hydroxyurea (HU) for 6 weeks while 9 were not (CON). HU mice displayed a higher specific total force production compared to the CON, with 501.35 ± 54.12 N/mm^3^ and 437.43 ± 57.10 N/mm^3^ respectively (+14.6%, *p* < 0.05). Neither the total rate of energy consumption nor the oxidative metabolic rate were significantly different between groups. The present results illustrated a positive effect of a HU chronic supplementation on skeletal muscle function in mice with mild anemia.

## 1 Introduction

Hydroxyurea, also known as hydroxycarbamide (HU) is a ribonucleotide reductase inhibitor, leading to a reduced availability of intracellular deoxynucleotide triphosphate which is needed for DNA synthesis. It reactivates γ-globin synthesis and consequently fetal hemoglobin (HbF) production (Cokic et al., 2003; Almeida et al., 2012).

HU is the most commonly used treatment in sickle cell disease (SCD) for decades (Platt et al., 1984; McGann and Ware, 2015). Indeed by inducing HbF production, HU contributes to reduce the abnormal sickle hemoglobin (HbS) proportion thereby alleviating symptoms of SCD patients i.e., anemia, vaso-occlusive crisis, acute chest syndrome and transfusion requirement (Charache et al., 1995). Interestingly, HU is also used in other types of anemias such as β-thalassemia major (Algiraigri et al., 2017, 2014) or E/β-thalassemia (Algiraigri and Kassam, 2017) in which anemia is expressed together with microcytosis (Rees et al., 1998; Muncie and Campbell, 2009). For these β-thalassemias, HU could also enhance HbF production thereby increasing total hemoglobin (Hb) level and reducing anemia (Fucharoen et al., 1996; Arruda et al., 1997; Singer et al., 2005). The corresponding improved effectiveness of erythropoiesis has been related to the decreased disease severity (Fucharoen et al., 1996; Singer et al., 2005; Algiraigri et al., 2017; Algiraigri and Kassam, 2017).

Severe anemia (<7 g/dl) is accompanied by multiple consequences of oxygen reduction. More particularly, anemia affects the oxygen-carrying capacity of blood with a recognized impact on oxygen supply and utilization by the active muscles which ultimately impairs exercise capacity (Woodson et al., 1978). In that respect, HU has been able to substantially enhance quality of life of patients (Fucharoen et al., 1996; Singer et al., 2005; Ballas et al., 2006; Algiraigri et al., 2017).

Interestingly, Algiraigri and colleagues reported that “the more severe the type of β-thalassemia, the milder the response to HU” (Algiraigri et al., 2014). From that point of view and considering 1) the relationship between oxygenation, muscle energetics and muscle function (including fatigue) (Woodson et al., 1978; Haseler et al., 1999), 2) the potential beneficial effects of HU on Hb level and local blood flow previously reported (Fucharoen et al., 1996; Cokic et al., 2003; Green and Barral, 2014), one could expect that a chronic HU supplementation could improve muscle energetics and function even in mild anemia. Interestingly, humanized hα/hα::βA/βA Townes mice exhibit a slightly lower Hb concentration compared to wild type mice (Wu et al., 2006) and thus constitutes an excellent model of mild anemia.

In the present study, we intended to assess the effects of a chronic HU supplementation on muscle energetics and function during a standardized rest-exercise-recovery protocol in a mild anemic murine model.

## 2 Methods

### 2.1 Animal Model, Care and Feeding

Seventeen humanized hα/hα::β^A^/β^A^ Townes mice of the same litter/lineage and two (±0.5) months old have been used in the present study (Jackson Laboratories, Bar Harbor, United States). These mice are characterized by a lower Hb concentration (mild anemia) and a reduced MCV as compared to wild type counterparts (Wu et al., 2006). The study was performed in conformity with the French guidelines for animal care and in conformity with the European convention for the protection of vertebrate animals used for experimental purposes and institutional guidelines n° 86/609/CEE 24 November 1986. All animal experiments were approved by the Institutional Animal Care Committee of Aix-Marseille University (permit number #1841-2019011016466656). Mice were housed in a controlled-environment facility (12–12 h light–dark cycle, 22°C) and received water and standard food *ad libitum*. Eight out of seventeen mice were supplemented with HU for 6 weeks (HU). Hydroxyurea was given every day (50 mg/kg/day) (Lebensburger et al., 2010) in drinking water and the concentration was adjusted daily according to the water consumption, we ensured that mice never lacked water. Previous studies have reported a positive effect of a similar dose provided acutely or for a 6 week-period on hematological characteristics (Platt et al., 1984; Lebensburger et al., 2010). Control mice (CON, *n* = 9) received water only over the same period. No animals died during the experiments.

### 2.2 Study Design

All mice were investigated after the 6-week period. Mice were anesthetized and weighted then muscle energetics and function were assessed in HU and CON mice in response to a standardized Rest-Stimulation (1 Hz)-Recovery protocol. One week later, mice were anesthetized in an induction chamber with isoflurane and then euthanized by cervical dislocation. Gastrocnemius muscle was harvested and weighted.

### 2.3 Non-Invasive Investigation of Posterior Hindlimb Muscles Function and Bioenergetics

Investigations were conducted using an innovative homebuilt experimental setup which has been designed to be operational inside the PharmaScan^®^ AVANCE™ III HD 70/16 US equipped with a 90 mm BGA09S (760 mT/m) gradient insert (Bruker BioSpin MRI Gmbh, Ettlinge, Germany). The setup allows 1) magnetic resonance imaging (MRI), 2) muscle force measurements and 3) ^31^P-magnetic resonance spectroscopy (^31^P-MRS) (Giannesini et al., 2010). MRI was used in order to get anatomical information about the hindlimb. The posterior hindlimb muscles mechanical performance was assessed with a dedicated homemade ergometer consisting of a foot pedal coupled to a force transducer (Giannesini et al., 2010). ^31^P-MRS was used to dynamically monitor the levels of high-energy phosphorylated compounds and acidosis. We have chosen to study posterior hindlimb muscles, which form the belly of the calf, because it is clearly distinct from the other muscles of the leg, easily accessible for MR coils and large enough to give ^31^P-MRS spectra with a good signal to noise ratio in a short time. It has been shown that the gastrocnemius muscle was preferentially activated using our experimental protocol (Giannesini et al., 2010).

### 2.4 Animal Preparation

Mice were anesthetized in an induction chamber (Equipement vétérinaire Minerve, Esternay, France) using an air flow (3 L/min) containing 4% isoflurane. The left hindlimb was shaved and electrode cream was applied at the knee and heel regions to optimize electrical contacts. The anesthetized animal was then placed supine in the experimental setup (Giannesini et al., 2010). Corneas were protected from drying by applying ophthalmic cream, and the animal’s head was placed in a facemask continuously supplied with 1-2% isoflurane mixed in 66% room air (1 L/min) and 33% O_2_ (0.5 L/min). Breathing rate was monitored during the protocol and kept between 100 and 130 breaths/min due to isoflurane concentration adjustment. The foot was positioned on the ergometer’s pedal and the hindlimb was centered inside a 16-mm-diameter ^1^H Helmholtz coil while the belly of the gastrocnemius muscle was located above an elliptic (8 × 6 mm^2^) ^31^P surface coil. Body temperature was controlled and maintained at a physiological level throughout the experiment using a feedback loop including an electrical heating blanket, a temperature control unit (ref. 507137, Harvard Apparatus, Holliston, MA, United States) and a homemade rectal thermometer.

### 2.5 Induction of Muscle Contraction and Contractile Force Measurement

Muscle contractions were induced by electrostimulation using two transcutaneous surface electrodes connected to a constant-current stimulator (DS7A, Digitimer, Herthfordshire, United Kingdom). One electrode was placed at the heel level and the other one was located just above the knee joint. Electrical signal coming out from the force transducer of the ergometer was amplified (Two operational amplifiers: AD620 and OP497, Analog Devices, Norwood, MA, United States, with a total dB gain: 70 dB at 0-5 kHz bandwidth) and continuously monitored and recorded on a personal computer using a Powerlab 16/36 data acquisition system (AD Instruments, Sydney, Australia) and a dedicated software (LabChart 8, AD Instruments, Sydney, Australia). The digital signal was converted to force according to a linear calibration curve and expressed in mN.

### 2.6 Moderate Exercise Protocol

Function and bioenergetics of the posterior hindlimb muscles were evaluated throughout a 6-min moderate exercise consisting of maximal isometric contractions repeated at a frequency of 1Hz, induced with square wave pulses (0.5 ms duration).

Force parameters were acquired with LabChart software (AD Instruments, Oxford, United Kingdom). Peak force (Pf, mN) was quantified. Total force production (TFP) was computed as the sum of each twitch. Specific values (Pf, TFP) were normalized to gastrocnemius muscle volume (cm^3^ or mm^3^) computed from anatomic hindlimb MR images.

### 2.7 Preliminary Adjustments

Before the onset of MR acquisition, the muscle was passively stretched at rest by adjusting the angle between the foot and the hindlimb to produce maximal twitch tension in response to supramaximal square waves pulses (0.5 ms duration). Individual maximal stimulation intensity was determined by a progressive increase of the stimulus intensity until there was no further force increase.

### 2.8 Multimodal MR Data Acquisition

Ten consecutive non-contiguous axial slices (1 mm thickness, spaced 0.25 mm) covering the region from the knee to the ankle were selected across the lower hindlimb. RARE images (Rare factor = 8, effective echo time = 35.29 ms, actual echo time = 11.76 ms, repetition time = 5,000 ms, one accumulation, 20 × 20 mm field of view, 256 × 192 matrix size, acquisition time = 3 min 14 s) were recorded at rest in order to assess muscle volume. Posterior hindlimb muscles region was manually delineated on each of the 6 largest slices. Then, muscle volume of each slice was extracted using the FSLeyes software (McCarthy, 2020). Total muscle volume (mm^3^) was calculated using the truncated cone volume formula considering slice and gap volumes.

Multiecho T2-weighted images (16 echo times equally spaced from 7.28 ms to 116.59 ms, 2000-ms repetition time, one accumulation, 20 × 30 mm field of view, 256 × 256 matrix size, total acquisition time = 8.32 min, slice thickness = 1 mm, slice gap = 1 mm) were recorded at rest. T2-weighted images were processed to generate T2-maps on a pixel by pixel basis by fitting the corresponding data with a single exponential function. Mean T2 values of posterior hindlimb muscles were measured on T2 maps and averaged on the 2 largest consecutive slices from the outlined regions of interest. The corresponding values were quantified in total gastrocnemius (GA) muscle, in the tibialis anterior (TA) region.

^31^P-MRS spectra (8 kHz sweep width; 2048 data points) from posterior hindlimb muscles were continuously acquired before (rest period; 4.7 min duration), during and after (recovery period; 16 min duration) the 6 min moderate exercise. Spectra acquisition was gated to muscle electrostimulation in order to reduce potential motion artifacts due to contraction. A total of 800 saturated free induction decays (FID, 1.875 s repetition time) were acquired. The first 140 FID were acquired in resting muscle and summed together. The next 450 FIDs were acquired during exercise and were summed as blocks of 15, allowing a 30 s temporal resolution. The remaining 210 FIDS were obtained during the recovery period and were summed as blocks of 30 FIDs.

### 2.9 MRS Data Processing

MRS data were processed using IDL-based (Interactive Data Language, Exelis, Visual Information Solutions, Boulder, CO,United States) custom-written routines (Le Fur et al., 2010) integrating the AMARES Fortran code (Vanhamme et al., 1997) from jMRUI (http://www.jmrui.eu/).

Relative concentrations of phosphocreatine (PCr), inorganic phosphate (Pi) and ATP were obtained from ^31^P-MRS spectra using the AMARES-MRUI-based time domain fitting routine including appropriate prior knowledge for the ATP multiplets. Absolute amounts of phosphorylated compounds were expressed taking into account a 5 mM β-ATP basal concentration (Giannesini et al., 2013). PCr relative concentration was expressed relative to the PCr content at rest which was set at 100%. Intracellular pH was calculated from the chemical shift of the Pi signal relative to PCr according to the formula (Arnold et al., 1984): pH = 6.75 + log [(3.27-δPi)/(δPi-5.69)]. Cytosolic ADP concentration was calculated, as previously described (Kemp et al., 1993; Harkema and Meyer, 1997): [ADP] = ([Cr] x [ATP])/([PCr]x10^−pH^ x K_CK_), using the creatine kinase equilibrium constant (K_CK_ = 1.66 × 10^9^ M^−1^) (Kemp et al., 2001a) and total creatine [Cr] calculated by assuming that PCr represents ∼85% of total Cr (Kemp et al., 2007). The rate of PCr degradation (VPCr_stim_, in mM/min) at the onset of exercise was calculated as VPCr_stim_ = ΔPCr/τPCr_stim_, where ΔPCr is the extent of PCr depletion measured at the exercise-end (relative to basal value) and τPCr_stim_ is the time constant of PCr degradation. τPCr_stim_ was determined by fitting the time course of PCr to a mono-exponential function using a least-means-squared algorithm. Force-normalized non-oxidative ATP synthesis (PCr_cost_ in µmol/N) was calculated as the ratio between VPCr_stim_ and the amount of force produced at the beginning of exercise, considering at that time that PCr is the only source of ATP production. Similarly, the PCr recovery kinetic parameters (τPCr_rec_) were determined during the post-exercise period by fitting the time course of PCr resynthesis to a mono-exponential function. Force-normalized oxidative ATP synthesis (PCr_rec_ in mM/N) was calculated as the ratio between VPCr_rec_ and the amount of force produced at the end of exercise. As previously suggested, VPCr_rec_ was considered as the rate of oxidative ATP production at end of exercise as VPCr_rec_ = ΔPCr/τPCr_rec_ (Boska, 1991; Prompers et al., 2014). The maximal rate of PCr recovery, V_max_, has been calculated as V_max_ = VPCr_rec_ (1 + (K_m_/[ADP_end_])), where K_m_ is the affinity constant of the mitochondria for ADP (30 μM in gastrocnemius muscle of the mice), and [ADP_end_] is the ADP concentration at the end of exercise.

### 2.10 Statistics

All values are presented as means ± SD except on graphics for which SE are displayed for the sake of clarity. Sample distribution was tested with the Shapiro-Wilk test and visual distribution (qqnorm plots). Significant differences were assessed using non-parametric Wilcoxon tests or Welch t-tests. All statistics were performed using R, on RStudio (4.0.2). The significance level was set at *p* < 0.05.

## 3 Results

### 3.1 Morphological Characteristics and Muscle Imaging

Body weight differed significantly between CON and HU groups. As indicated Table 1, HU mice were heavier (34.26 ± 1.94 g) than CON (30.91 ± 2.83 g, *p* = 0.013). Otherwise, relative and absolute gastrocnemius weights and volumes did not differ between groups.

**TABLE 1:** Muscles and body weights in HU and CON mice.

	CON (*n* = 9)	HU (*n* = 8)
Body weight (g)	30.91 ± 2.83	34.26* ± 1.94
Gastrocnemius weight (mg)	156.59 ± 24.36	158.31 ± 15.62
Gastrocnemius weight/body weight (mg/g)	5.09 ± 0.79	4.62 ± 0.33
Muscle volume (mm^3^)	154.76 ± 15.82	158.93 ± 15.41

Results are presented as means ± SD. *significantly different from CON.

T2 values of the gastrocnemius were significantly lower in HU (29.43 ± 0.84 ms) as compared to CON (30.79 ± 1.70 ms, p = 0.0328, Table 2). Regarding the tibialis anterior, T2 values were not significantly different between HU (25.33 ± 0.55 ms) and CON (25.74 ± 0.63 ms) groups (*p* = 0.0952, Table 2).

**TABLE 2:** T2 values in muscles of HU and CON mice.

	CON (*n* = 9)	HU (*n* = 8)
Tibialis anterior	25.74 ± 0.63	25.33 ± 0.55
Whole GA	30.79 ± 1.70	29.43 ± 0.84 *

Results are presented as means ± SD. * significantly different from CON.

### 3.2 Posterior Hindlimb Muscles Bioenergetics

**FIGURE 1:**
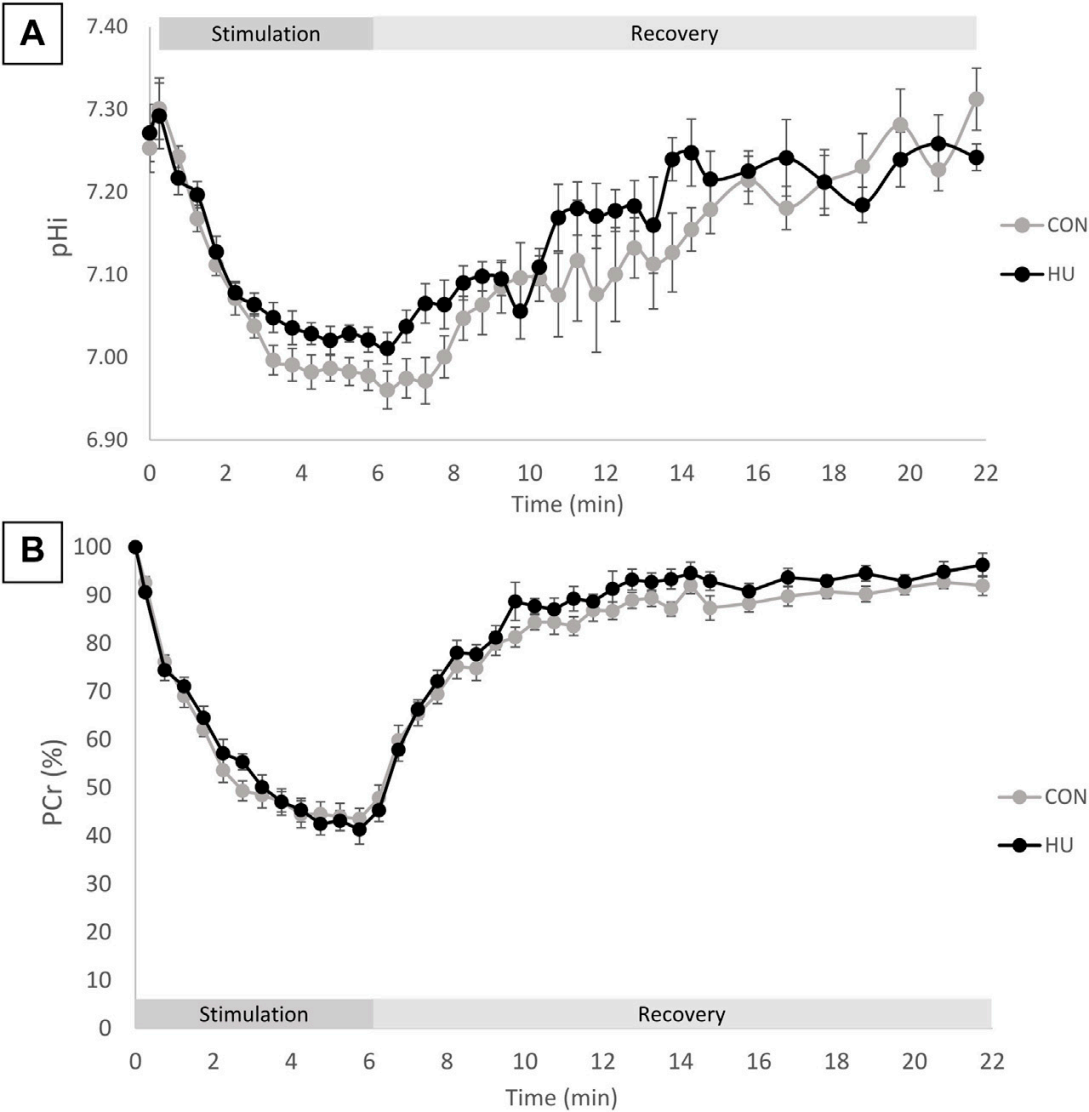
**(A)** pHi time course during the rest-stimulation-recovery protocol in CON and HU. Results are presented as means ± SEM. **(B)** Relative PCr time-course during the rest-stimulation-recovery protocol in CON and HU. Results are presented as means ± SEM.

Intracellular pH at rest was similar in HU (7.27 ± 0.09) and CON (7.25 ± 0.08) mice (*p* = 0.6765, Table 3). During the 6-min moderate-intensity stimulation period, a transient pH_i_ elevation was observed, followed by a pH_i_ reduction. This reduction occurred in both groups with an initial faster decrease followed by a slower phase (Figure 1A). At the end of the exercise period, pH_i_ decreased of 0.25 ± 0.10 and 0.28 ± 0.09 pH unit in HU and CON group respectively (*p* = 0.58). The initial recovery phase started in a steady-state and continued with a progressive increase towards the resting value (Figure 1A). At the end of the recovery period, pH_i_ was not significantly different in HU and CON (7.24 ± 0.04 and 7.31 ± 0.11, respectively, *p* = 0.0953). The end of exercise ADP was not significantly different between HU (55.17 ± 12.06 μM) and CON (48.94 ± 8.46 μM, *p* = 0.23, Table 3) groups.

**TABLE 3:** Metabolic index at rest and exercise.

	CON (*n* = 9)	HU (*n* = 8)
pHi_rest_	7.25 ± 0.08	7.27 ± 0.09
pHi_end_	7.31 ± 0.11	7.24 ± 0.04
PCr depletion (%)	−56.45 ± 6.14	−58.68 ± 8.12

Results are presented as means ± SD. No significant variation was found.

As illustrated in Figure 1B, the relative PCr time course can be divided in 3 phases. An initial progressive decrease occurred during the first 4 min of exercise, followed by a plateau from minute 4 to 6 reaching a depletion of −58.68% ± 8.12 and −56.45% ± 6.14 for HU and CON group respectively (*p* = 0.53, Table 3). The recovery period was characterized by a progressive return towards the baseline values.

The rate of PCr degradation (VPCr_stim_) and the rate of PCr resynthesis (VPCr_rec_) were not significantly modified between HU (4.82 ± 1.25 mM/min and 3.69 ± 1.28 mM/min, respectively) and CON (5.44 ± 1.03 mM/min and 3.08 ± 0.61 mM/min, respectively) group (*p* = 0.2924 and *p* = 0.2512).

The time constant of PCr degradation (τPCr_stim_) was not significantly different between HU (2.03 ± 0.90 min) and CON (1.57 ± 0.39 min) groups (*p* = 0.2109). The time constant of PCr resynthesis (τPCr_rec_) was similar in HU (2.12 ± 0.43 min) and CON (2.19 ± 0.42 min) groups (*p* = 0.7459).

**FIGURE 2:**
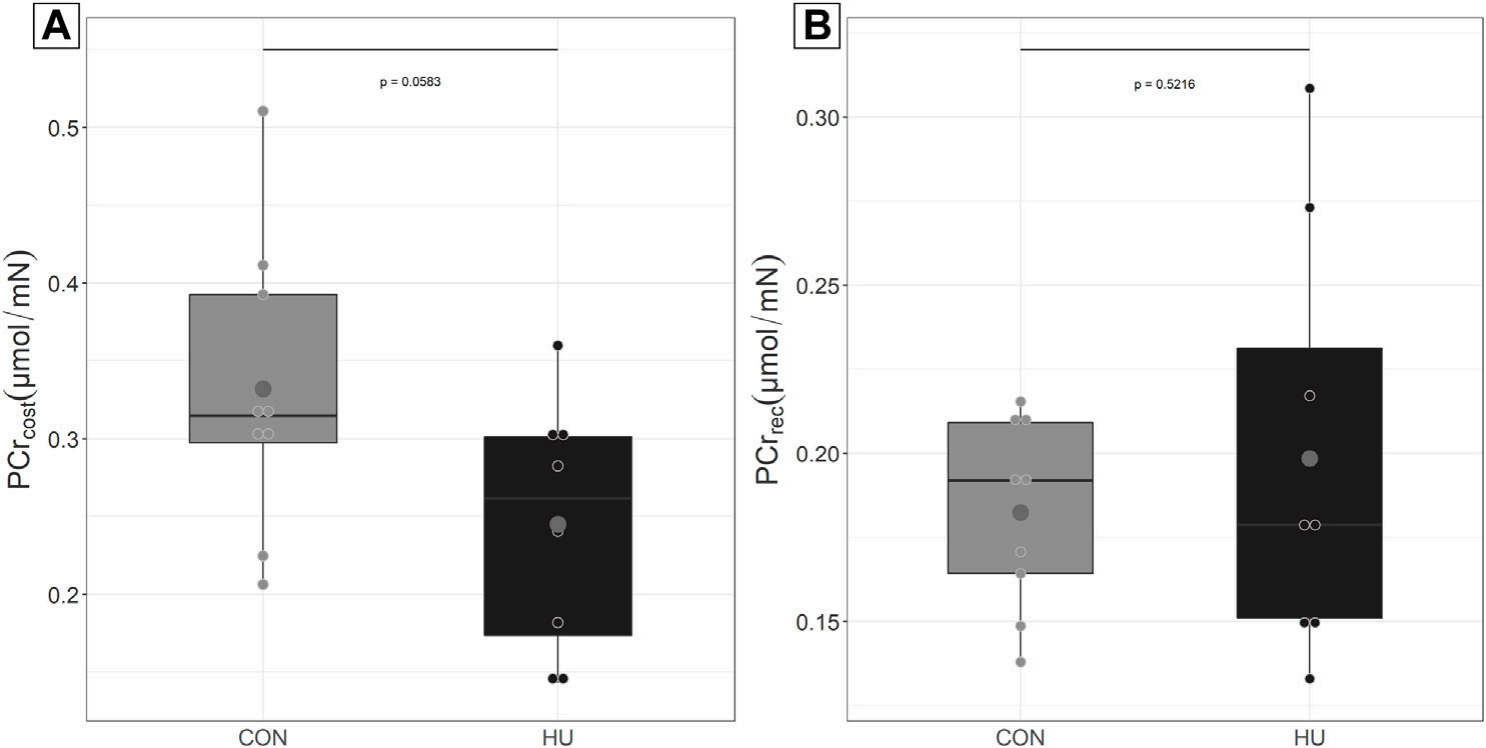
**(A)** PCr_cost_ at the beginning of the moderate exercise in CON (grey, *n* = 9) and HU (black, *n* = 8). results are presented in boxplot. **(B)** PCr_rec_ at the end of moderate exercise, results are presented in boxplot.

The force-normalized non-oxidative ATP synthesis i.e., PCr_cost_ strongly tended to be lower in HU compared to CON (Figure 2A, *p* = 0.0583). The force-normalized oxidative ATP synthesis i.e. PCr_rec_ was similar in HU and CON mice (Figure 2B, *p* = 0.5216).

The maximal rate of PCr recovery, V_max_, was similar (*p* = 0.3) between CON (4.99 ± 0.86 mM/min) and HU (5.68 ± 1.68 mM/min) groups.

### 3.3 Muscle Function

**FIGURE 3:**
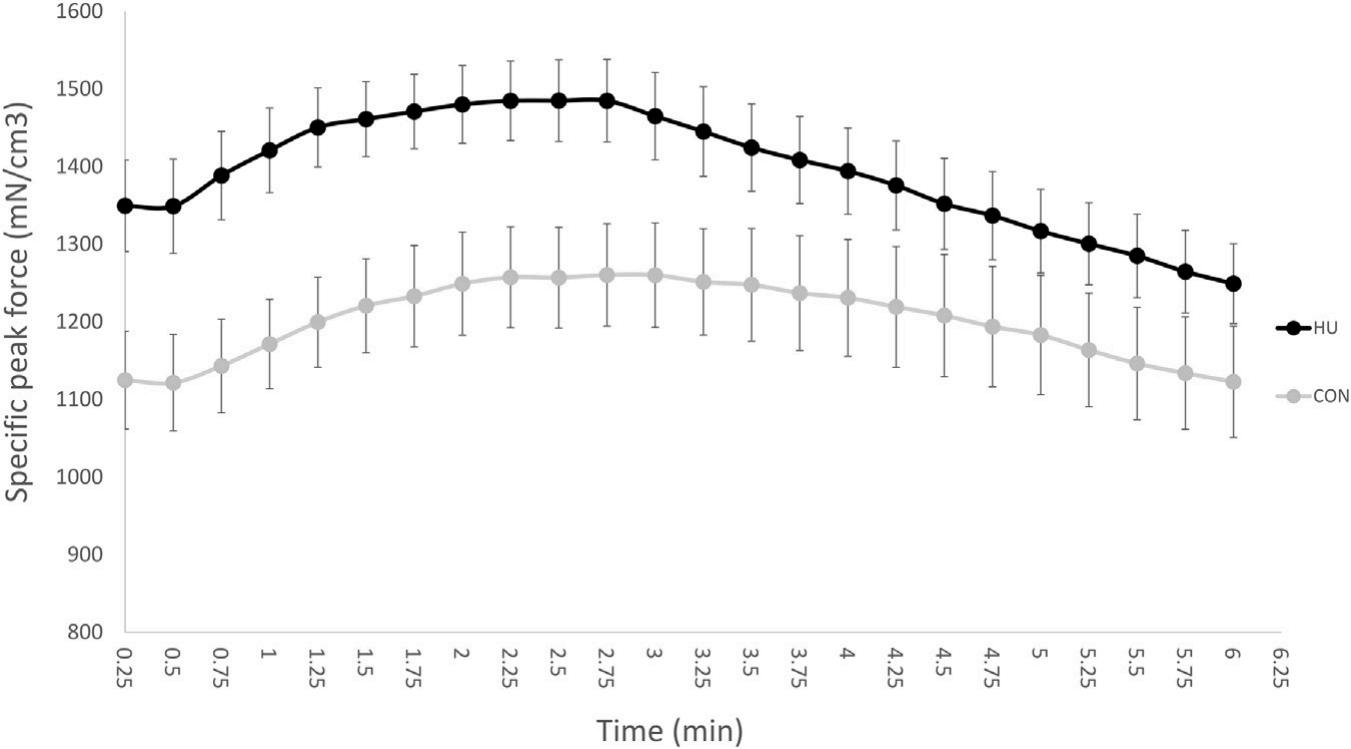
Time dependent changes in specific peak force throughout the moderate exercise in CON (grey, *n* = 9) and HU-treated mice (black, *n* = 8). Results are presented as means ± SEM.

At the onset of stimulation, specific peak force was significantly higher in HU mice (1,348.17 ± 165.82 N/cm^3^) as compared to CON mice (1,121.78 ± 140.78 N/cm^3^, *p* = 0.0093, Figure 3).

As illustrated in Figure 3, specific peak force time-course was biphasic with an initial increase and a peak reached after a 90 s stimulation period. Afterwards, specific peak force progressively decreased until the end of stimulation.

**FIGURE 4:**
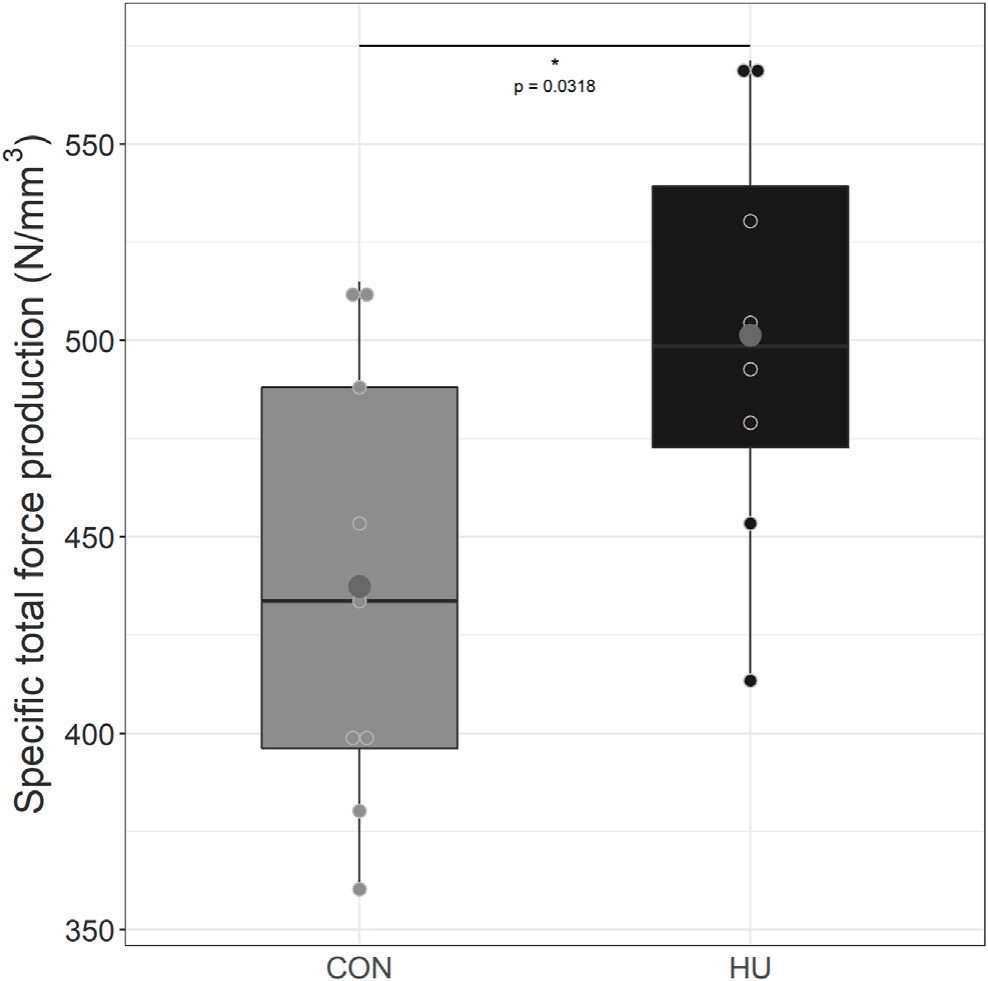
Specific total force production from during the 6 minutes moderate exercise in CON (grey, *n* = 9) and HU (black, *n* = 8) groups, results are presented in boxplot. ^*^ significantly different from HU group.

As illustrated in Figure 4, HU mice (501.35 ± 54.12 N/mm^3^) produced a significantly larger specific total force production over the 6-min stimulation period as compared to controls (437.43 ± 57.10 N/mm^3^, *p* = 0.0318).

## 4 Discussion

In the present study, we aimed at characterizing the changes occurring in skeletal muscle energetics and function in response to a 6-week chronic hydroxyurea supplementation in mildly anemic mice. Our results showed that HU supplementation 1) strongly tended to enhance PCr_cost_, 2) decreased posterior hindlimb averaged T2 values and 3) increased specific total force production.

### 4.1 Effect of HU on Skeletal Muscle Oxygenation

Considering the well-known effect of HU on fetal Hb production, we hypothesized that HU could improve muscle oxidative capacity as a consequence of an increased oxygen delivery. This hypothesis was supported by the fact that a beneficial effect of HU treatment has been established on aerobic capacities. Indeed, sickle cell anemia (SCA) patients receiving HU decreased their heart rate in response to a standardized exercise compared to untreated SCA patients (Hackney et al., 1997). The authors suggested an improved cardiovascular efficiency without mentioning underlying mechanisms. In addition, HU supplementation has been related to an increased NO bioavailability which can have a vasorelaxant effect and improve muscle oxygenation (Chen et al., 2008; Roberson and Bennett-Guerrero, 2012; Green and Barral, 2014).

As pH and ADP concentration were similar at the end of exercise, the initial rate of PCr resynthesis, VPCr_rec_ should reflect V_max_ as previously suggested (Roussel et al., 2000). Therefore, only VPCr_rec_ will be discussed. In the present study, the unchanged rate of PCr resynthesis, VPCr_rec_, quantified during the post-stimulation period did not support this hypothesis. The rate of PCr resynthesis and the corresponding kinetic metrics have been largely acknowledged as illustrative of muscle oxidative function (McCully et al., 1993; Kemp and Radda, 1994; Conley et al., 2000; Praet et al., 2007; Lanza et al., 2011). In addition, it has been shown that PCr recovery kinetics could be modulated by the oxygenation status. More specifically, PCr recovery kinetics can be accelerated in hyperoxic conditions in a trained population (Haseler et al., 1999), whereas hypoxia can reduce the corresponding rate in a sedentary and trained population (Haseler et al., 2007, 2004).

Along the same line, it has been previously reported that patient with peripheral arterial disease (PAD) who suffer from a decreased skeletal muscle O_2_ supply displayed a significant reduced PCr recovery kinetics (Kemp et al., 2001b). Of interest, it has been reported that hyperoxia was not sufficient to improve the recovery kinetics in PAD patients (Hart et al., 2018). However, an improved mitochondrial function was observed when hyperoxia was combined to reactive hyperemia (Hart et al., 2018) thereby suggesting that both convective and diffusive O_2_ supply may limit mitochondrial oxidative capacity in PAD patients (Hart et al., 2018). These results are totally supportive of the present results obtained in anemic conditions. While HU is expected to increase oxygen supply in the same way as hyperoxia in PAD patients, we could suggest that the unchanged PCr recovery kinetics in HU-treated mice is indicating that anemic muscle is limited not only by oxygen convection but also by oxygen diffusion to muscle cells.

One can also wonder if the lack of effect of HU in the present study may be related to the low impact of mild anemia observed in the used model on the muscle oxidative capacity and mitochondrial function. Moreover, it cannot be excluded that the lack of difference may be related to compensatory mechanisms affecting cardiac output and microvascular blood flow could occur in mild anemic conditions thereby limiting the deleterious effects of the reduced oxygen delivery (Roberson and Bennett-Guerrero, 2012). Overall, these compensatory changes would account for the similar oxidative capacity in treated and untreated mice. Additional measurements are warranted in order to decipher the exact mechanisms.

Nevertheless, PCr_cost_, an index of the non-oxidative ATP production, tended to be reduced by HU supplementation (*p* = 0.0583). This can be understood as a reduced glycolytic contribution and/or a larger oxidative production in exercising muscle which could support our primary hypothesis. It would have then to be determined why a potential improved oxygenation could modulate the aerobic ATP production during exercise and not during the post-exercise recovery phase.

### 4.2 Effect of HU on the ATP Cost of Contraction

The ATP cost of contraction represents the rate of ATP utilization for a given muscle activity. The rate of ATP utilization is actually supported by both oxidative and non-oxidative contributions. In the present study and as previously mentioned, PCr_cost_ strongly tended to be reduced in HU mice compared to CON counterparts (*p* = 0.058). These results support those from Bailey *et al* who obtained a reduced PCr consumption in humans during an incremental knee-extension test after beetroot juice (a nitrate provider) supplementation (Bailey et al., 2010).

This decreased PCr_cost_ can be understood as a lower rate of ATP utilization for a given force or a faster rate of ATP resynthesis which mainly relies on oxidative capacity. In the study of Bailey *et al*, the PCr_cost_ reduction was explained as a reduced rate of ATP hydrolysis for a given work (Bailey et al., 2010). Of note, ATP demand is conditioned by both contractile and non-contractile processes keeping in mind that non-contractile processes such as sarcoplasmic Ca^2+^ pumping can represent up to 20-50% of the total ATP turnover (Bergstrom and Hultman, 1988).

Nevertheless, a faster oxidative rate of ATP resynthesis by oxidative pathway cannot be totally excluded. Ferguson et al (2015) reported a higher microvascular partial pressure of oxygen (PO_2_mv) after a dietary nitrate supplementation (Ferguson et al., 2015) which could be expected to increase the rate of oxidative wing of ATP production thereby reducing the exercise-induced PCr consumption (Haseler et al., 1998; Vanhatalo et al., 2011; Ferguson et al., 2015). Neither the present results nor those from Bailey et al (2010) indicated an improved rate of PCr resynthesis, VPCr_rec._

Moreover, oxidative stress, which has been reported as elevated in various anemias (Akohoue et al., 2007; Fibach and Rachmilewitz, 2008; Nur et al., 2011; Voskou et al., 2015; Vinhaes et al., 2020), has been previously reported to impair mitochondrial function (Halestrap et al., 1993; Guo et al., 2013). Of interest, it has been previously reported that SCA patients treated with HU displayed a reduced degree of oxidative perturbation as compared to untreated SCA patients (Vinhaes et al., 2020). If such a phenomenon was occurring in HU-treated mice, our results indicate that it did not affect mitochondrial function.

The reduced PCr_cost_ could result from a reduced rate of ATP hydrolysis. Accordingly, Hernandez *et al* suggested that the larger force production resulting from nitrate ingestion would result from changes in Ca^2+^ handling (Hernández et al., 2012), indicating that the non-contractile ATP demand might be reduced.

### 4.3 Effect of HU on Force Production

Specific total peak force (total peak force scaled to muscle volume) developed by HU-treated mice was significantly higher as compared to CON. Such a direct effect of HU on force production has never been reported in mild anemic mice. However, Wali & Moheeb reported a higher absolute force production of the right hand and leg in HU-supplemented children compared to healthy controls (Wali and Moheeb, 2011). HU has also been associated to a decreased heart rate during a treadmill exercise together with a longer exercise time before exhaustion both indicative of an improved exercise capacity (Wali and Moheeb, 2011). A similar beneficial effect on exercise capacity has already been described in HU-treated SCA patients with an improved performance for the Wingate test (Hackney et al., 1997). Both results have been directly related to the increased fetal hemoglobin level with a corresponding reduction in the HbS level and a reduction of clinical symptoms.

Of interest, *in vitro* measurements could suggest some accounting factors of the force increase. An increased contractile force has been reported *in vitro* in fast-twitch fibers (dominant fiber type) in mice supplemented with nitrate, an important NO precursor (Hernández et al., 2012). Of interest, HU supplementation has been shown to increase NO production and bioavailability (Jiang et al., 1997; Glover et al., 1999; Huang et al., 2002). More specifically, HU can oxidize oxy and deoxy-hemoglobin to methemoglobin, which can then react with other HU molecules and form nitrosylhemoglobin (HbNO) slowly releasing NO (Jiang et al., 1997).

Alternatively, this increased force production might be related to a larger relative amount of fast-twitch fibers in HU mice. This means that HU would induce a slow to fast-twitch fiber transition. The reduced T2 values we quantified in HU mice compared to CON in the present study might be supportive of this hypothesis since lower T2 values have been related to a larger quantity of fast-twitch glycolytic fibers (Hatakenaka et al., 2001). The averaged T2 of water protons illustrates the molecular environment together with the water mobility. Previous studies have mainly reported T2 changes (mainly increase) in a variety of conditions such as eccentric exercise, inflammatory disorders etc. (Fisher et al., 1990; Marqueste et al., 2008; Esposito et al., 2013; Bryant et al., 2014; Zaccagnini et al., 2015), and reduced T2 values have been scarcely reported.

Considering the beneficial effects of HU in daily activity of patients (Fucharoen et al., 1996; Singer et al., 2005; Ballas et al., 2006; Algiraigri and Kassam, 2017), cage activity of mice in the present study might have increased thereby accounting for the improved muscle function (Bowden Davies et al., 2019).

### 4.4 Effect Mediated by Nitric Oxide

Overall, HU-supplementation was related to a likely reduced non oxidative cost of contraction and an increased skeletal muscle force production. On the basis of literature results and of the well-known effects of HU on NO production, we strongly pointed out the increased NO bioavailability as a strong potential accounting factor for the present results. Although NO measurements would have to be performed as a confirmatory support, potential underlying effects mediated by NO are of interest to mention.

The increased bioavailability of NO would improve local blood flow, fatigue resistance and fiber contractility (Jones et al., 2016). In this way, the increased contractility may have increased force production and decreased the cost of non-contractile processes, inevitably reducing ATP consumption.

## 5 Conclusion

In the present study, we observed a potential ergogenic effect of a chronic supplementation of hydroxyurea in a group of mice with mild anemia. HU supplementation improved force production, modified bioenergetics and reduced the average T2 values. Further studies are necessary to confirm the underlying mechanisms of the observed changes.

## Data Availability

The raw data supporting the conclusions of this article will be made available by the authors, without undue reservation.
